# Sentinel Lymph Node Biopsy and Complete Lymph Node Dissection for Melanoma

**DOI:** 10.1007/s11912-019-0798-y

**Published:** 2019-04-26

**Authors:** Alberto Falk Delgado, Sayid Zommorodi, Anna Falk Delgado

**Affiliations:** 1 Department of Plastic Surgery, Uppsala University, Ing 85, Akademiska Sjukhuset, 75185 Uppsala, Sweden; 2 Department of Molecular Medicine and Surgery, Karolinska Institutet, Stockholm, Sweden; 3 Department of Plastic Surgery, Karolinska University Hospital, Stockholm, Sweden; 4 Clinical neurosciences, Karolinska Institutet, Stockholm, Sweden; 5 Department of Neuroradiology, Karolinska University Hospital, Stockholm, Sweden

**Keywords:** Melanoma, Sentinel node, Biopsy, Complete, Lymph node, Limb perfusion, Dissection, Survival, Overall survival, Outcome, Surgery, Review, Metastasis, Therapy, Regional, Early, Surgical oncology, Surgical margin

## Abstract

**Purpose of Review:**

The main surgical treatment for invasive malignant melanoma consists of wide surgical and examination of the sentinel node and in selected cases complete lymph node dissection. The aim of this review is to present data for the optimal surgical management of patients with malignant melanoma.

**Recent Findings:**

A surgical excision margin of 1–2 cm is recommended for invasive melanoma depending on the thickness of the melanoma. Sentinel node biopsy may be considered for patients with at least T1b melanomas thickness 0.8 to 1.0 mm or less than 0.8 mm Breslow thickness with ulceration, classified as T1b lesion, per recent AJCC guidelines. Two randomized controlled trials have been published—DeCOG (German Dermatologic Cooperative Oncology Group Selective Lymphadenectomy) and MSLT-2 (Multicenter Selective Lymphadenectomy Trial) comparing the complete lymph node dissection (CLND) with observation after positive sentinel node biopsy. In the MSLT-2 study, the disease control rate was improved in the immediate CLND group compared with observation but there was no difference in 3-year melanoma specific survival (86% ± 1.3% and 86% ± 1.2%, respectively; *p* = 0.42). Isolated limb perfusion (ILP) or isolated limb infusion (ILI) with melphalan and actinomycin D is recommended for large and multiple in-transit metastases and satellite metastases in the extremities when local excision is considered ineffective or too extensive.

**Summary:**

In light of new adjuvant treatment options and new indications for checkpoint inhibitors, and the lack of survival benefit after CLND, we can expect open surgery to decrease in melanoma disease.

## Introduction

There is a rising incidence of melanoma and the expected incidence of cutaneous melanoma in the USA is 91.270 cases 2018 with 22 new cases per 100.000, constituting approximately 5 % of all cancer cases according to Surveillance, Epidemiology, and End Results (SEER) National Cancer Institute [1]. There has been a substantial improvement in the 5-year overall survival over the last decades; from 81% in 1970 to 92% 2008–2014. While 5-year survival in localized disease is 98%, survival from disease with distant metastasis is much lower; 22%.

Malignant melanoma is characterized by high mutation rates, higher than most cancer types. Large efforts have been directed towards describing the genomic landscape in melanoma disease, which has been divided into four genetic subclasses: *BRAF* mutations, *RAS* mutations, mutant *NF1*, and triple *WT* (wild-type) [2]. More recent studies have shown that acral and mucosal melanomas can lack mutations in *TP53*, *PTEN*, and *RB1*, as well as having lower mutation rates. This suggests a distinct molecular etiology for acral and mucosal compared with cutaneous melanomas [3].

The general main surgical treatment for invasive malignant melanoma consists of wide surgical excision with clear histological margins and removal and examination of the sentinel node—the first drained lymph node to be affect by metastatic disease—to detect occult disease for staging and prognosis [4], and in selected cases complete lymph node dissection. The aim of this review is to present data for the optimal surgical management of patients with malignant melanoma.

### Treatment

#### Surgical Excision

The standard treatment of melanoma is wide and radical excision including deep tissue. If the resected margins are not clear from malignant melanoma at histological examination, any remaining melanoma cells in the surrounding tissue should be included in a re-excision. Surgical margins are based on the maximal melanoma Breslow thickness (measured in millimeters) of the melanoma [5]. All pigmented lesions with a clinical suspicion of melanoma should be removed with at least 2-mm clinical clear margin, but not exceeding 5 mm to preserve the lymphatic drainage assessed by sentinel node biopsy (SNB) at a later stage [6, 7]. Excision of lesions located on the extremities should follow the length axis to facilitate primary closure and avoidance of skin grafts. For the removal of invasive (as defined by histology) melanoma, the excision should continue through the skin and subcutaneous tissue down to, but not including, the fascia/periosteum/perichondrium. For melanoma in situ, surgical excision should include the superficial subcutaneous tissue [6]. Partial biopsies of suspected melanomas should be avoided due to the risk of under staging, but if necessary can be guided by dermatoscopy to identify the most malignant part of the melanoma suspected lesion [8]. Importantly, the risk of sentinel lymph node metastasis or overall survival has not been associated with the choice of biopsy method (excisional versus incisional versus shave biopsy) [9].

#### Timing

In a study from the National Cancer Database (*N* = 153.218), data suggested that surgery performed later than 90 days was associated with a higher mortality for melanoma of all stages [10]. Furthermore, in a subgroup of patients with stage T1(less than 1-mm thick melanoma) disease, patients had higher mortality if surgery was delayed > 30 days [10].

#### Surgical Margins

For melanoma in situ, a clinical margin of 5 mm is considered sufficient to obtain a histological clear margin. This was originally based on an expert consensus statement in 1993 [11]; however, new data recommends wider excision for obtaining clear histological margins [12]. In contrast, no data supports extended surgical margins if histological free margins have already been achieved. In case of ambiguity, discussion in a multidisciplinary conference is recommended.

For thin but invasive melanomas (less than 1 mm), a 1-cm surgical margin is considered a sufficient margin [13–15]. This margin is based on three randomized control trials (RCTs) which have used at least a 1-cm margin [16–19]. A meta-analysis conducted by the Cochrane Collaboration and published in 2009 concluded that there were insufficient data to make a clinical recommendation of excision margins for thin melanomas [20]. For intermediate and thick melanomas, six RCTs comparing narrow (1–2 cm) and wide excision (3–5 cm) [16, 17, 19, 21–30] have been published. A recent meta-analysis found no difference in overall survival (HR 1.09; 95% CI 0.98–1.22; *p = 0.1*, six trials) between the groups, nor in loco-regional recurrence (HR 1.10; 95% CI 0.96–1.26; *p = 0.2*, six trials). However, in a subgroup analysis including four trials only reporting on melanoma specific survival wide excision was favored HR 1.17 (95% CI 1.03–1.34; *p = 0.02*). There are currently two trials registered in ClinicalTrials.gov randomizing melanoma patients to 1- or 2-cm surgical margins for melanomas thicker than 1 mm or T2 melanoma (> 1.00–2.00 mm) [31, 32].

Surgical excision can in almost every case be performed under local anesthesia. For the lower extremities, local flaps such as the keystone flap [33] are common for cover after wide excision but should not be undertaken if histological free margins have not yet been achieved.

#### Contraindications

In clinical practice, there are few contraindications for performing primary excision. Refraining from primary excision can be due to patient declining surgery or presenting with a very poor overall health condition.

### Surgery

#### Sentinel Node Biopsy

Sentinel node biopsy (SNB) is the surgical procedure where the sentinel lymph node is removed and investigated for the presence of cancer cells. SNB was developed in order to identify early metastases in regional lymph nodes and to select only patients with nodal metastases to undergo complete lymph node dissection and avoiding this in patients without nodal metastases [34]. The sentinel node SN is identified using lymphoscintigraphy after the injection of radioactive Technetium adjacent to the primary tumor, and in many sites in combination with blue dye injected preoperatively in four quadrants around the primary site. Perioperative detection of the SN is then accomplished with a gamma probe and by ocular inspection of blue dye in the operation field. SNB has a false negative rate of approximately 10–20% [35–37]. In addition, SNB for melanoma in the axilla is considered more challenging than in breast cancer as the SN is often located deeper in the axilla in level II-III. Indocyanine green is a new method of mapping the lymph node without using radioactive tracer and without the risk of tattooing that comes with the use of blue dye [38]. Further, injection with paramagnetic nanoparticles in combination with blue dye has shown comparable results as Technetium with blue dye in detecting the SN in breast cancer; however, this has not been tested for melanoma [39]. Sentinel node biopsy without the need of a radioisotope does not require a Nuclear medicine department, which could make the sentinel node procedure possible even in smaller hospitals.

The SNB is used for staging of melanoma disease and is an independent prognostic factor besides tumor thickness [40, 41]. SNB may be considered for patients with T1b melanomas thickness 0.8 to 1.0 mm or less than 0.8 mm Breslow thickness with ulceration, classified as T1b lesion, per the recent AJCC 8th edition [42]. A positive SN has been found in approximately 5.2% of thin melanomas (≤ 1 mm) [43] and in 8% of melanomas thicker than 0.8 mm [44]. Ulceration is associated with increased risk for SN positivity [43], while there is little supporting evidence that mitoses in thin melanomas are associated with SN positivity [43, 45]. Therefore, SNB should be offered for T1b lesion (0.8–1.0 mm) or less than 0.8-mm lesions with ulceration. Informing the patient of the SNB procedure with its associated risks and benefits should always forego any surgical treatment.

One RCT has compared SNB with nodal observation (clinical examination), in the 10-year follow-up with 2001 participants; there were 340 thin melanomas (< 1.2 mm), 1347 intermediate-thickness melanomas (1.2–3.5 mm), and 314 thick melanomas (> 3.5 mm) [46•]. Patients were randomized to SNB and subsequent complete lymph node clearance (if SNB positive) or nodal observation and complete lymph node dissection if clinical nodal relapse. There was no difference in 10-year follow-up in melanoma specific survival for intermediate-thickness melanoma and thick melanomas (HR 0.84; 95% CI, 0.64–1.09; *p* = 0.18) and (HR 1.12; 95% CI, 0.76–1.67; *p* = 0.56). DFS was favored in the SNB group compared with nodal observation HR 0.76 (95% CI, 0.62–0.94; *p* = 0.01) and HR 0.70 (95% CI, 0.50–0.96; *p* = 0.03). In patients with nodal metastases in intermediate-thickness melanoma, the SNB group had a lower risk for death than the observation group (HR 0.56; 95% CI, 0.37 to 0.84; *p* = 0.006). This was however not seen in the group with thick melanomas. The overall risk for SN positivity is approximately 14–20% in intermediate-thickness melanomas [4, 47, 48]. Given the improved disease control and role of staging for intermediate-thickness melanomas (1–4 mm), SNB is recommended in these patients.

For thick melanomas, (> 4 mm), SNB is recommended mainly for staging and for potential disease control, it is important to point out that this particular patient group is at higher risk of systemic disease; therefore, the therapeutic benefit is perhaps more limited. In certain cases of very thick melanomas, we conduct imaging a priori to surgery since this could lead to substantial changes in the surgical treatment [49].

#### Localization of the Sentinel Node

Most upper extremity melanomas drain to the axillary basin while lower extremity melanomas usually drain to the inguinal basin. Melanomas of the trunk have a more unpredictable draining pattern and might drain to either or both basins. SNB for melanomas in the head and neck region is somewhat more complex [50] and multiple lymph node basins can be affected. For distal lower extremity melanoma, 3–9% [51, 52] of patients present with a dual-basin drainage (popliteal fossa and the inguinal basin). Lymph nodes along the course of a lymphatic vessel between the primary melanoma site and the recognized basin are sometimes referred to as an interval node. The role of interval nodes in the surgical approach for SNB remains insufficiently studied but not removing them has been suggested to increase the risk of undetected metastatic disease [53].

#### Contraindications

High age is not an absolute contraindication for SNB but severe comorbidities as, e.g., dementia is often a relative contraindication for performing SNB in clinical practice; therefore, an individual evaluation of the patient’s health is warranted. The benefit of SNB in systemic disease is probably very limited; therefore, this is often a contraindication for performing the SNB.

#### Complications to SNB

The complication rate has been reported at 10% in the MSLT-1 study consisting mostly of seroma/hematoma and infections [54]. Lymph edema has been reported to occur in 1.5–1.7% of SNB cases [55].

### Complete Lymph Node Dissection

Complete lymph node dissection (CLND) has been a cornerstone in the management of melanoma patients with a positive SNB for many years. The underlying idea behind performing CLND is to prevent the melanoma disease from systemic spreading and attain accurate staging.

Two RCTs have been published—DeCOG (German Dermatologic Cooperative Oncology Group Selective Lymphadenectomy) [56•] and MSLT-2 (Multicenter Selective Lymphadenectomy Trial) comparing the CLND with observation after positive SNB [57••]. DeCOG included patients between 18 and 75 years with tumor thickness of at least 1 mm; 22 patients were included with a tumor thickness less than 1 mm, and with micrometastasis in the SN (head and neck melanomas were excluded). DeCOG was stopped prematurely due to problems of recruiting patients, and hence the study finished underpowered and did not find any differences in survival. MSLT-2 included patients between 18 and 75 years of age and SN-positive patients were randomized to either CLND or observation and CLND only in patients with nodal recurrence [57••]. The disease control rate was improved in the immediate CLND group compared with observation but there were no difference in 3-year melanoma specific survival (86% ± 1.3% and 86% ± 1.2%, respectively; *p* = 0.42). One issue with the two RCTs is related to their inclusion within 120–140 days after a positive SNB, suggesting that there might be a time benefit that goes undetected in these studies with relatively long time to surgery and no reported subgroup analysis between early and late surgery [58]. Furthermore, most patients in the study had small tumor burden, approximately 10–12% were only detectable on RT-PCR and not seen in the microscope, and invasion of the lymph node capsule in the SNB was an exclusion criteria. However, a subgroup with larger nodal tumor burden in the sentinel node compared with small tumor burden did not indicate a survival advantage. In addition, in both De-COG and MSLT-2 patients underwent serial nodal ultrasounds; thus, lack of this resource might limit generalizability of these studies.

In the meta-analysis by Falk Delgados including four RCTs, comparing immediate CLND with observation/delayed CLND there was no survival benefit from CLND [59]. However, melanoma-specific survival was higher after immediate CLND compared with delayed CLND in patients with nodal metastasis (HR = 0.63, 95% CI = 0.35–0.74, *p* = 0.0004) without evidence of heterogeneity in the analysis, suggesting there is a time-dependent disease-specific survival in early/immediate lymph node surgery. This could explain why there is no additional survival benefit after immediate SNB/CLND compared with performed at a later stage, suggesting that the surgical benefit for a patient with regional MM metastases lies not in the procedure itself (SNB/ CLND) but in its timing. In clinical practice, it is challenging to determine which patients should undergo CLND or not. It is unclear if CLND in situations of more advanced disease in the axilla for, e.g., periglandular growth or several positive SN has any additional therapeutic effect. Considering the new era of adjuvant treatment, both COMBI-AD [60••] and CHECK-MATE 238 [61••] trials included stage III or IV melanoma, with patients required to undergo CLND before randomization to systemic treatment or placebo. It is unclear if there is a benefit with CLND compared with observation in combination with adjuvant treatment such as BRAF/MEK inhibition or PD-1 inhibition. In the case of a positive SNB, an honest and open discussion with the patient is needed where the risks and the benefits of the procedure need to be accounted for. Furthermore, in case of nodal recurrence without signs of distant metastases CLND can be considered. As CLND becomes less common, surgical expertise to perform the procedure will probably decrease with time, this will demand that the surgical procedure is concentrated to certain specialized centers.

#### Complications

The MSLT-2 study reported a 24% rate of lymphedema after CLND compared with 6% in the observation group [57••]. De Vries et al. reported a worse Quality of Life after CLND compared with SNB only [62]. In the meta-analysis by Moody et al. the total complication rate after CLND was 37.3% (21.6% infection/delayed wound healing, 18% lymphedema, 17.9% seroma, and 1.5% hematoma) [63].

### Isolated Limb Perfusion and Isolated Limb Infusion

Isolated limb perfusion (ILP) or isolated limb infusion (ILI) with melpahalan and actinomycin D is recommended for large and multiple in-transit metastases and satellite metastases in the extremities when local excision is considered ineffective or too extensive [64]. ILI is considered less invasive and can be repeated more easily than ILP. Both ILI and ILP are effective treatments of locally advanced disease. Clinical overall responses have been reported to 81–90% after ILP and 41–53% after ILI; however, there is no randomized controlled trial comparing ILP and ILI [65–67]. Despite the higher response rates with ILP compared with ILI, there could be a selection biased towards offering ILI to older patients. It would be of interest to see the effectiveness of new systemic treatment in combination with ILP/ILI for the treatment of locally advanced melanoma disease. Aryian et al. report the results from 26 patients with advanced melanoma treated with ILP and CTLA-4 inhibition with a response rate of 85% [68].

#### Complications

Approximately 40–60% of patients report transient neuropathy after ILP and ILI [69]. Most of the patients report acute complications such as erythema and blistering as a direct result of the perfusion treatment; however, approximately 2% report extensive epidermolysis [65]. Common reactions to ILP are mild or severe erythema.

#### Future Perspectives

We expect more accurate diagnosis of dermatological diseases including that of melanoma using smartphone applications and artificial intelligence probably leading to an increased detection rate of early stage melanoma [70].

In terms of performing a more narrow excision, currently, the MelMar study is recruiting patients with invasive melanoma ≥ 1 mm and randomizing them to 1 cm or 2-cm excision margins The results of this study might lead to more precise surgical margin recommendations than the current standard of care [31].

The role of CLND, even in the presence of regional nodal recurrence, will be debated. It is clear from our clinical experience that CLND has decreased the last years, and in the future this procedure will be best performed in specialized melanoma centers.

In light of new adjuvant treatment options and new indications for checkpoint inhibitors where studies are designed to treat patients with regional or local advanced disease, we can expect open surgery to decrease.

FDG-PET is today’s gold standard to assess distant melanoma metastases [71]. However, the main problem with FDG-PET is the availability of scanners and nuclear tracers. Further, since glucose metabolism is not specific for malignant disease other nonmalignant disease processes can imitate metastatic disease and hamper correct diagnosis [72]. Further, FDG-PET in treated patients have a risk of false negative findings and limited detection of micrometastatic disease [73] that in the future could be alleviated using more specific tracers directed towards a narrowly defined disease process. Further, in the advent of development of very fast MRI scanning, enabling full body MRI scans in 1–2 min, this has the potential of becoming the new standard imaging modality [74].
